# Animal welfare in deep-bedded pig systems: A systematic review, evidence synthesis, and translational frameworks for sustainable veterinary practice

**DOI:** 10.14202/vetworld.2026.3683-3700

**Published:** 2026-08-27

**Authors:** Kulisara Marupanthorn, Watcharapong Wattanakul, Korawan Sringarm, Chaiwat Arjin, Niraporn Chaiwang

**Affiliations:** 1 Division of Animal Sciences, Faculty of Agricultural Technology, Chiang Mai Rajabhat University, Chiang Mai 50300, Thailand.; 2 Department of Animal and Aquatic Sciences, Faculty of Agriculture, Chiang Mai University, Chiang Mai, Thailand.

**Keywords:** animal welfare, deep-bedding, evidence synthesis, meta-analysis, pig production, rice husk, sustainable livestock production, veterinary medicine

## Abstract

**Background and Aim:**

Deep-bedded housing systems have been increasingly adopted as alternatives to conventional pig production because they provide manipulable substrates, improve behavioral opportunities, and support circular manure management. However, their effects on animal welfare, production performance, and environmental sustainability remain inconsistent across production stages, bedding materials, and management systems. Furthermore, evidence comparing individual bedding materials is fragmented, particularly for tropical substrates such as rice husk. This systematic review aimed to critically evaluate the effects of deep-bedded pig systems on animal welfare, productivity, environmental sustainability, and economic performance through qualitative evidence synthesis and quantitative meta-analysis, and to develop practical translational frameworks to support veterinary decision-making.

**Materials and Methods:**

A systematic review was conducted according to the PRISMA 2020 guidelines using Web of Science, Scopus, and PubMed to identify studies published between January 2000 and March 2026. A total of 790 records were retrieved, of which 221 comparative studies met the eligibility criteria for qualitative synthesis. Pairwise random-effects meta-analyses were performed for average daily gain (ADG), feed conversion ratio (FCR), and mortality when sufficient quantitative data were available. Risk-of-bias was assessed using Risk-of-bias In Non-randomized Studies of Interventions (ROBINS)-I and RoB 2 principles, whereas certainty of evidence was evaluated using the Grading of Recommendations Assessment, Development and Evaluation. The feasibility of network meta-analysis was also examined. Two translational frameworks, the Healthy Pig Production Framework and the Sustainable Deep-Bedding Model, were developed to integrate scientific evidence into veterinary practice.

**Results:**

Deep-bedding consistently improved behavioral welfare by increasing rooting, exploratory activity, and resting comfort compared with conventional housing. Meta-analysis demonstrated no overall effect on ADG (mean difference: +9.8 g/day; 95% confidence interval: −58.3 to 77.9; p = 0.78), although subgroup analysis indicated improved growth in nursery-growing pigs and reduced performance in growing-finishing pigs. Evidence for FCR and mortality remained inconclusive because of incomplete reporting and substantial heterogeneity. Environmental outcomes indicated improved manure recycling potential but greater risks of ammonia emissions and greenhouse gas production when bedding moisture and ventilation were poorly managed. Material-specific evidence was insufficient to support a formal network meta-analysis, highlighting a major research gap. Rice husk emerged as a promising bedding material for tropical production systems, although current evidence remains predominantly qualitative.

**Conclusion:**

Deep-bedded housing should be regarded as a management-dependent strategy for welfare and sustainability rather than a simple flooring alternative. Although welfare benefits were consistently observed, production and environmental outcomes varied by production stage, substrate type, and management quality. The proposed translational frameworks provide practical tools for veterinary assessment and sustainable pig production while identifying priorities for future substrate-specific comparative research.

## INTRODUCTION

Animal welfare is increasingly treated as a core quality criterion in pig production, alongside productivity, food safety, and environmental accountability. The Five Freedoms and the Five Domains Model frame welfare as more than the absence of disease: pigs also require behavioral opportunity, physical comfort, thermal suitability, and conditions that support positive affective states. Deep-bedding systems, also termed deep-litter, bedded pack, or substrate-based systems, offer a biologically plausible route to these aims because straw, sawdust, wood shavings, rice husk (rice hull), and mixed organic substrates can support rooting, nosing, resting comfort, and manure-solidification processes that are not available on bare concrete or fully slatted floors [1–4].

The significance of this review lies in the mismatch between the theoretical welfare promise of deep-bedding and the fragmented evidence available for veterinary and farm decision-making. Previous work has often treated productivity or environmental outcomes as the main endpoint, whereas welfare outcomes, substrate-specific evidence, tropical applicability, and rice husk systems in rice-producing regions have received less integrated attention. This gap matters for One Health, circular economy, and regional policy discussions because deep-bedding can reduce liquid effluent and create recoverable spent litter, yet it can also increase ammonia, dust, heat load, or hygienic risk if the bedding process is poorly managed. Therefore, material choice cannot be separated from production stage, climate, ventilation, stocking density, and farm labor capacity [5–15].

The aim of this study was to conduct a welfare-centered systematic review and evidence-network synthesis of deep-bedded pig systems. The specific objectives were (1) to summarize welfare, health, production, environmental, and economic outcomes across the eligible literature; (2) to estimate pooled production effects where extractable average daily gain (ADG), feed conversion ratio (FCR), or mortality data allowed quantitative synthesis; (3) to test whether a material-specific network meta-analysis (NMA) was feasible and, if not, to report the disconnected evidence-network transparently; and (4) to develop two novel translational tools, the Healthy Pig Production Framework (HPPF) and the Sustainable Deep-Bedding Model (SDBM), to support veterinary audit, producer decision-making, and future research design.

## MATERIALS AND METHODS

### Ethical approval

This systematic review used published and publicly available study-level data and did not involve new animal or human subjects. Ethical approval and informed consent were therefore not required.

### Study period and location

The literature search, data extraction, statistical analysis, and interpretation of the findings were conducted in March 2026. All stages of the review were conducted at Chiang Mai University, Chiang Mai, Thailand.

### Study design

This systematic review and meta-analysis followed PRISMA 2020, and the attempted network component was reported in line with PRISMA-NMA where applicable. The review question, eligibility criteria, extraction variables, and planned syntheses were finalized before data extraction. This registration limitation is stated transparently, and the final protocol logic, curated dataset, PRISMA flow values, screening logs, and analysis files are available in the Open Science Framework repository and accompanying supplementary files. The study period covered literature published from January 2000 to March 2026 [16, 17].

### PICOS framework

**This review used PICOS to define eligibility and analysis. Population (P):** domestic pigs (*Sus scrofa domesticus*) at any production stage, including farrowing/breeding, nursery/weaner, growing, finishing, and mixed-stage systems. Intervention (I): deep-bedding or deep-litter systems using organic substrates, including straw, sawdust, wood shavings, rice husk/rice hull, or mixed substrates; no single universal depth threshold was imposed for qualitative eligibility because source studies defined deep-bedding inconsistently, but reported depth or bedding amount was extracted where available. Comparator (C): conventional non-bedded housing, slatted or concrete floors, outdoor/pasture comparators, or direct material comparisons. Outcomes (O): primary production outcomes were ADG, FCR, and mortality; secondary outcomes included behavior, injuries, lameness, hygiene, thermal comfort, respiratory indicators, emissions, wastewater, compost/nutrient recovery, economics, and welfare-audit indicators. Study design (S): randomized and non-randomized comparative studies were eligible; case reports, opinion articles, and single-arm descriptions without extractable comparator information were excluded or used only as background (Table 1).

**Table 1:** Eligibility criteria and analytical framework used in the integrated evidence synthesis.

**Domain**	**Specification**	**Operational note**
Population	Domestic pigs at any production stage	Farrowing, nursery, growing, finishing, and mixed-stage production systems were eligible.
Intervention	Deep-bedding and deep-litter housing	Eligible substrates included straw, sawdust, wood shavings, rice husk (rice hull), and mixed organic materials. No universal bedding depth threshold was applied because definitions varied among studies.
Comparator	Conventional non-bedded housing or direct bedding material comparison	Pairwise meta-analysis compared deep-bedding with conventional housing, whereas the network component required direct head-to-head comparisons between bedding materials.
Primary outcomes	ADG, FCR, and mortality	ADG was quantitatively synthesized by meta-analysis. FCR and mortality were narratively synthesized because numerical data were insufficient for quantitative pooling.
Secondary outcomes	Welfare, health, environmental, and economic outcomes	Outcomes included exploratory behavior, injuries, lameness, thermal comfort, hygiene, respiratory health, emissions, wastewater generation, compost value, economic performance, and welfare-audit indicators.
Study designs	Randomized and non-randomized comparative studies	Case reports, opinion papers, conference abstracts, reviews, and studies without an appropriate comparator were excluded.
Analytical components	Systematic review, pairwise meta- analysis, network meta-analysis feasibility assessment, ROBINS-I, GRADE, HPPF, and SDBM	The review comprised a systematic review, pairwise meta-analysis, assessment of network meta-analysis feasibility, risk-of-bias assessment using ROBINS-I, certainty assessment using GRADE, and development of the HPPF and SDBM.

ADG = Average daily gain; FCR = Feed conversion ratio; GRADE = Grading of Recommendations Assessment, Development and Evaluation; HPPF = Healthy Pig Production Framework; NMA = Network meta-analysis; ROBINS-I = Risk-Of-Bias In Non-randomized Studies of Interventions; SDBM = Sustainable Deep-Bedding Model.

### Search strategy

A comprehensive search was conducted in Web of Science Core Collection, Scopus, and PubMed/MEDLINE. The final database strings combined pig terms (pig, swine, porcine, hog, sow, gilt, boar, piglet, weaner, grower, finisher), housing/substrate terms (bedding, deep-litter, straw, sawdust, wood shavings, rice husk, substrate, floor type, flooring, housing), and outcome terms (welfare, behavior/behavior, lameness, injury, tail, stereotypy, performance, growth, emissions, ammonia, greenhouse gas, environment, manure, compost). Field tags were adapted to each platform: TS= in Web of Science, TITLE-ABS-KEY in Scopus, and Title/Abstract tags in PubMed. The complete platform-specific Boolean strings, date limits, and syntax are provided in Supplementary Table S1. No formal gray-literature search or expert-contact program was undertaken; reference lists and locally available full texts were checked where accessible, and the absence of formal gray-literature searching is acknowledged as a limitation.

### Eligibility criteria and study selection

Study selection proceeded through title/abstract screening and full-text assessment by two independent investigators using predefined eligibility criteria. Disagreements were resolved through discussion and, where needed, a third investigator. The final PRISMA/open science flow was: 790 records identified (Web of Science 312, Scopus 289, PubMed 189), 565 duplicate records removed, 225 reports screened and assessed for eligibility, 0 reports not retrieved, 4 records removed during final duplicate/eligibility reconciliation, and 221 studies retained for qualitative synthesis. Five study/comparison entries had sufficient ADG information for pairwise meta-analysis, and one complete straw-versus-sawdust comparison was available for direct material-specific analysis.

Inclusion criteria were original comparative pig studies published from January 2000 to March 2026; domestic pigs at any production stage; at least one deep-bedding/deep-litter or named organic bedding treatment; conventional housing, outdoor/pasture, or material comparison group; and at least one welfare, health, production, environmental, or economic outcome. Exclusion criteria were non-pig studies, reviews or opinion papers without primary data, single-arm reports without a comparator, duplicate reports, and reports lacking information needed for the intended synthesis. Non-English records were not excluded at search retrieval, but extractability depended on available English metadata, translations, or accessible full-text; this language/extractability constraint is reported as a limitation. The quantitative subset is summarized in Table 2.

For material-oriented extraction, substrates were standardized as straw, sawdust/wood shavings, rice husk/rice hull, mixed organic bedding, or unspecified deep-litter. Rice husk and rice hull were treated as synonymous terms. Records that lacked exact per-arm means, Standard deviation (SD)/ Standard error (SE), sample size, or duration were retained for qualitative synthesis but excluded from pooled numeric analysis unless effect estimates could be transparently reconstructed from reported values.

**Table 2:** Characteristics of studies contributing to the quantitative synthesis.

**Reference**	**Country**	**Production ** **stage**	**Comparison**	**Sample size**	**Key quantitative information**	**Contribution to synthesis**
[30]	Belgium	Weaned pigs	Straw vs. sawdust	200 vs. 200	ADG: 397 ± 40 vs. 402 ± 28 g/day; FCR: 1.80 ± 0.04 vs. 1.79 ± 0.04; mortality: 2.5 ± 0.9% vs. 2.5 ± 0.9%	Only complete direct bedding material comparison; used for the direct material-specific analysis.
[27]	Brazil	Growing-finishing	Deep-bedding vs. conventional housing	NR	ADG: 779 vs. 824 g/day; SD not reported	Included in the stage-specific qualitative interpretation of ADG.
[28] (small pigs)	China	Nursery- growing	Deep-bedding vs. conventional housing	18 vs. 18	ADG increased by 4.52%; FCR improved by 3.16% in the deep-litter group	Included in the pooled ADG meta-analysis.
[28] (large pigs)	China	Nursery- growing	Deep-bedding vs. conventional housing	16 vs. 16	ADG increased by 16.73%; FCR improved by 7.43% in the deep-litter group	Included in the pooled ADG meta-analysis.
[26] (Producer A)	Commercial farm	Growing-finishing	Deep-bedding vs. conventional housing	NR	ADG: −0.05 kg/day; FCR: +0.15 kg/kg in the deep-bedding group	Included in the stage-specific qualitative interpretation of ADG.
[26] (Producer B)	Commercial farm	Growing-finishing	Deep-bedding vs. conventional housing	NR	ADG: −0.03 kg/day; FCR: +0.05 kg/kg in the deep-bedding group	Included in the stage-specific qualitative interpretation of ADG.
[29]	Colombia	Finishing	Rice husk deep-bedding vs. concrete floor	100 vs. 40	Mortality: 4.0% vs. 0.0%; no significant differences in ADG or FCR	Only study providing extractable mortality data for quantitative comparison.

NR = Not reported in the original study. Only studies contributing directly to the quantitative synthesis or the direct bedding material comparison are included.

### Data extraction

Data from each eligible article was independently extracted into a structured Google Sheets workbook and checked against the curated OSF dataset. Extracted fields included author(s), year, country, climate, production stage, genetics where reported, substrate type, litter depth or bedding amount, comparator, pen or pig sample size, duration, outcome definitions, ADG, FCR, mortality events, welfare and health indicators, environmental indicators, economic findings, missing-data notes, and risk-of-bias domains. Data management used Google Sheets and Microsoft Excel; quantitative synthesis tables were prepared in R (version 4.3.0, meta and metafor packages) and cross-checked against Review Manager 5.4.1 outputs where applicable.

For continuous outcomes, means were extracted with SD when available; SE or 95% confidence interval (CI) values were converted to SD using standard formula (for example, SD = SE × √n) when sample size was reported. Percentage changes, direction-only findings, and p-value-only reports were not treated as fully extractable raw data unless a defensible effect estimate was already available in the curated extraction tables. Missing-data and conversion decisions were documented in the supplementary extraction and QC files. Inter-rater reliability statistics (for example, Cohen's kappa) were not available in the extraction logs, so this article reports the independent dual-investigator process and consensus resolution rather than adding an unsupported reliability value.

For quantitative meta-analysis, studies were required to report or support reconstruction of treatment and comparator values with an interpretable variance estimate. Studies reporting only qualitative direction, percentage change without sufficient denominator information, or significance statements were retained in narrative synthesis. Complete extractable raw data and reasons for exclusion from quantitative pooling are summarized in the supplementary tables and OSF files.

### Meta-analysis

A minimum of three independent studies or study/comparison entries with extractable numeric data was required for a pooled pairwise outcome. Random-effects models were used because production stage, climate, substrate, genotype, housing bundle, and management intensity were expected to differ across studies. ADG and FCR were analyzed as mean differences (MD) using consistent units (g/day and kg feed per kg gain, respectively); mortality was analyzed as a risk ratio where event data were available. The primary τ² estimator in the curated analysis was DerSimonian–Laird, with fixed-effect and leave-one-out sensitivity analyses reported where feasible.

ADG was expressed as MD (g/day) between deep-bedding and conventional housing. FCR was expressed as MD (kg/kg), where lower values indicate better feed efficiency. Mortality was expressed as risk ratio (RR) for deep-bedding relative to conventional housing when event counts were available. Key findings are summarized in Table 3.

A priori subgroup analysis was performed by production stage (nursery-growing versus growing-finishing) because stage was the most biologically plausible modifier and had enough data for comparison. Additional exploratory subgroup summaries by study quality and geographic region are provided in the supplementary files. Formal meta-regression for climate, substrate, bedding depth, or management intensity was not performed because the number of extractable studies was too small and key covariates were sparsely reported.

**Table 3:** Summary of quantitative findings across pairwise and direct material-specific comparisons.

**Outcome/comparison**	**Studies (participants)**	**Effect estimate**	**Heterogeneity/certainty**	**Interpretation**	**Practical meaning**
ADG: Deep-bedding vs. conventional housing (overall)	5 studies (>1,200 pigs)	MD = +9.8 g/day (95% CI = −58.3 to 77.9)	I² = 85.9%; Q = 28.5, p < 0.001; τ² = 3247; 95% prediction interval = −112.5 to +132.1; Very low	No overall difference	Average effect masks substantial contextual heterogeneity.
ADG: Nursery-growing subgroup	2 studies (~400 pigs)	MD = +87.0 g/day (95% CI = +12.0 to +162.0)	I² = 78%; Low	Favors deep- bedding	Suggests benefit when behavioral enrichment and thermal buffering coincide.
ADG: Growing-finishing subgroup	3 studies (~800 pigs)	MD = −41.7 g/day (95% CI = −52.4 to −31.0)	I² = 45%; Low	Favors conventional housing	Indicates a potential trade-off in heavier pigs or under warmer conditions.
FCR: Deep-bedding vs. conventional housing	6 studies (>1,000 pigs)	Qualitative synthesis only; extractable pooled estimate not defensible	Very low	Mixed pattern	Insufficient complete numerical reporting for pooled estimation.
Mortality: Deep- bedding vs. conventional housing	1 study (140 pigs)	RR = 3.63 (95% CI = 0.20 to 65.84)	Very low	Very uncertain	Wide CI prevents reliable inference.
ADG: Sawdust vs. straw	1 study (400 pigs)	MD = +5.0 g/day (95% CI = −1.8 to +11.8)	Low	Little or no difference	Any growth advantage of sawdust appears minimal.
FCR: Sawdust vs. straw	1 study (400 pigs)	MD = −0.01 kg/kg (95% CI = −0.018 to −0.002; p = 0.0128)	Moderate	Favors sawdust slightly	Approximately 1% improvement in feed efficiency in nursery pigs.
Mortality: Sawdust vs. straw	1 study (400 pigs)	MD = 0.0% (95% CI = −0.18 to +0.18)	Low	No meaningful difference	Mortality was identical in the direct-comparison.
Rice husk systems (qualitative synthesis only)	5 studies (sample sizes variably reported)	Narrative pattern = ADG and FCR were generally similar to concrete flooring, whereas mortality findings were inconsistent	Not pooled; Low	Promising but unranked	Rice husk appears operationally viable in tropical systems, but the available evidence is insufficient for formal ranking.

ADG = Average daily gain; CI = Confidence interval; FCR = Feed conversion ratio; MD = Mean difference; RR = Risk ratio.

Negative MD values for FCR favor the first-listed treatment because lower feed conversion ratios indicate better feed efficiency.

### Assessment of heterogeneity

Heterogeneity was evaluated with I^2^, τ², Cochran's Q, forest-plot inspection, sensitivity analysis, and the 95% prediction interval where estimable. For the overall ADG analysis, heterogeneity was high (I^2^ = 85.9%; Q = 28.5, p < 0.001; τ² = 3247; 95% prediction interval −112.5 to +132.1 g/day), so pooled estimates were interpreted as context averages rather than universal farm-level predictions. 

Publication bias was explored qualitatively using funnel-plot inspection and Egger's regression test as an underpowered screen, given that fewer than 10 studies contributed to the quantitative outcome. Egger's test for ADG was not significant (p = 0.24). Begg's rank-correlation test was not interpreted as a formal decision criterion because the number of studies was below the recommended threshold; the funnel plot was therefore treated as descriptive only. Selective outcome reporting remained plausible because only a small fraction of the 221-study evidence base reported complete variance data.

### NMA feasibility assessment

For the network component named bedding materials and direct material comparisons were mapped before analysis. A full NMA required at least a connected evidence-network with more than one directly or indirectly linked material comparison, sufficient outcome overlap, and no structural inconsistency that would make transitivity implausible. These criteria were not met; only one complete direct material comparison (straw-versus-sawdust) was available, while rice husk, wood shavings, mixed bedding, and generic deep-litter nodes were disconnected or lacked complete outcome data. Therefore, no treatment ranking or SUCRA-style hierarchy was calculated; the disconnected network is reported as a primary evidence gap.

### Assessment of publication bias and study quality

Two investigators independently extracted and verified data, and disagreements were resolved by consensus, with input from the third investigator when required. Risk-of-bias was assessed using Risk-of-bias in Non-randomized Studies of Interventions (ROBINS)-I for non-randomized studies and RoB 2 principles for randomized comparisons. Main-domain judgments covered confounding, participant selection, intervention classification, deviations from intended interventions, missing data, outcome measurement, and selective reporting. Table 4 reports the studies that directly contribute to the quantitative synthesis, while the supplementary ROBINS-I summary reports the broader qualitative evidence base [18, 19]. ROBINS-I domains assessed were confounding, participant selection, intervention classification, deviations from intended interventions, missing data, outcome measurement, and selection of reported results.

Certainty of evidence was appraised with the certainty of the evidence was assessed using the Grading of Recommendations, Assessment, Development and Evaluation (GRADE) approach. Because most included evidence was observational or non-randomized, certainty was commonly downgraded for risk-of-bias, inconsistency, imprecision, and suspected selective reporting. The supplementary GRADE table lists downgrade rationales for ADG, FCR, mortality, and the direct straw-versus-sawdust comparison [20, 21].

**Table 4:** ROBINS-I judgments for studies contributing directly to the quantitative synthesis.

**Reference**	**Confounding**	**Selection**	**Classification**	**Deviations**	**Missing-data**	**Measurement**	**Reporting**	**Overall**
[30]	Moderate	Low	Low	Low	Low	Low	Moderate	Moderate
[27]	Serious	Moderate	Moderate	Moderate	Moderate	Moderate	Serious	Serious
[28]	Serious	Serious	Serious	Moderate	Serious	Moderate	Serious	Serious
[26]	Serious	Serious	Moderate	Serious	Moderate	Moderate	Serious	Serious
[29]	Moderate	Moderate	Moderate	Moderate	Low	Moderate	Moderate	Moderate

ROBINS-I = Risk-of-bias In Non-randomized Studies of Interventions.

### Development of translational frameworks

Two translational constructs were developed (Table 5) after synthesis through iterative author discussion and consensus. The HPPF integrates the Five Domains model with practical veterinary audit indicators: behavioral fulfilment, physical integrity, physiological comfort, hygienic stability, and stage-matched productivity. The SDBM treats deep-bedding as a managed biophysical and circular-resource process linking substrate input, dietary nitrogen load, barn design, labor-management intensity, bedding moisture, aeration, microbial turnover, emissions control, and compost/nutrient recovery. These newly developed translational tools are intended to guide audit, implementation, and future validation rather than to serve as fully validated scoring systems. Their operational decision points are summarised in Table 6.

**Table 5:** Integrative synthesis of welfare, environmental, and economic outcomes in deep-bedding systems.

**Domain**	**Typical direction in deep-bedding systems**	**Key modifiers**	**Practical implication**	**Evidence certainty**
Exploratory behavior and behavioral opportunity	Improved	Substrate friability, replenishment, space allowance	Deep-bedding strongly supports rooting, nosing, and exploratory behavior.	Moderate-high (qualitative)
Physical comfort, lameness, and surface-related injury	Usually improved	Bedding dryness, compression, hygiene	Soft lying surfaces can reduce claw and limb injuries, but benefits decline when bedding becomes wet or compacted.	Low-moderate
Thermal comfort	Stage- and climate-dependent	Pig age, climate, ventilation, bedding heat load	Young pigs may benefit from insulation, whereas heavy pigs in warm conditions may experience greater thermal burden.	Low
Respiratory and hygienic stability	Variable	Dustiness, moisture, ventilation, microbial management	Health benefits are management-dependent; poor bedding management may increase dust-related risk.	Low
Ammonia emissions	Often higher than conventional systems	Nitrogen loading, moisture, aeration, additives	Emission mitigation is essential if the system is to remain environmentally sustainable.	Moderate
Greenhouse gas profile	Mixed	Methane reduction vs. nitrous oxide increase	Deep-bedding may reduce methane while increasing nitrous oxide emissions; therefore, the net environmental effect should be evaluated at the system level.	Low-moderate
Wastewater and manure circularity	Improved circularity	Substrate quantity, compost handling, crop integration	Reduced liquid effluent and recoverable spent- litter are major strengths of the system.	Moderate
Economic viability	Context-dependent	Bedding cost, labor, market premium, compost value	Economic outcomes depend heavily on local resource availability and production stage.	Low
Rice husk as a regionally relevant substrate	Potentially favorable but context-dependent	Moisture control, litter depth, regional availability, compost handling	Rice husk may be a practical substrate in rice-producing regions, but it should be managed as a process-sensitive material rather than assumed to be inherently superior.	Low

**Table 6:** Translation of the Healthy Pig Production Framework and Sustainable Deep-Bedding Model into operational decision points.

**Framework element**	**Core question**	**Operational indicator**	**Action point for practice**
HPPF - behavioral fulfilment	Can pigs perform sustained exploratory behavior?	Time spent rooting, nosing, digging; manipulation of substrate	Maintain fresh, friable substrate and adequate space.
HPPF - physical integrity	Does the floor protect the pig's body and limbs?	Lameness, bursitis, claw lesions, skin abrasions	Monitor leg and claw condition; prevent wet, compacted bedding.
HPPF - physiological comfort	Is the thermal and resting environment stage-appropriate?	Panting, huddling, lying pattern, body cleanliness	Adjust ventilation and bedding depth according to age and climatic conditions.
HPPF - hygienic stability	Is microbial and manure load under control?	Moisture, odor, coughing, fly density, pen hygiene	Replenish and aerate bedding; remove local wet patches promptly.
HPPF - stage-matched productivity	Is the system supporting the intended production stage?	ADG, FCR, days to market, culling/mortality	Use stage-specific nursery and finishing performance targets rather than assuming one system suits all production stages.
SDBM - substrate input	Is the bedding material locally appropriate?	Absorbency, carbon content, dustiness, cost, local availability, rice husk structure, litter depth	Prioritize locally available substrates with acceptable moisture control and handling properties; rice husk may be especially suitable in rice-producing regions when litter depth and replenishment are well managed.
SDBM - process-control	Is the composting/manure process stable?	Bedding temperature, moisture, addition frequency, aeration	Treat bedding as a managed biophysical process rather than as a passive floor covering.
SDBM - emissions control	Are environmental externalities being minimized?	Ammonia, odor, greenhouse gas indicators	Use additives, dietary nitrogen management, and appropriate ventilation where needed.
SDBM - circular output	Does spent-litter become a resource?	Compost maturity, nutrient value, crop utilization pathway	Link housing decisions to manure reuse on cropland or compost markets.

ADG = Average daily gain; FCR = Feed conversion ratio; HPPF = Healthy Pig Production Framework; SDBM = Sustainable Deep Bedding Model.

## RESULTS

### Characteristics of the evidence base

The 221 included studies covered farrowing/breeding, nursery/weaner, growing, finishing, and mixed-stage systems. Production stage information was available for 177 studies (80.1%). Finishing, growing, and nursery/weaner systems were the most common extractable stages, whereas evidence for breeding and farrowing was more fragmented. Named bedding information was available for all 221 studies: most records involved generic deep-bedding/deep-litter, while straw, sawdust/wood shavings, and rice husk/rice hull were the most common named materials. Country and climate information was sparse (57/221 studies, 25.8%), which limited formal climate-based synthesis.

Total animal numbers across the qualitative evidence base could not be reliably calculated because per-arm sample sizes were reported in only a small minority of records. This incompleteness is a central result rather than a clerical gap; it explains why the review could include 221 studies qualitatively but only five study/comparison entries in the ADG meta-analysis. The tables therefore separate the broad qualitative evidence base from the smaller quantitative subset and report missingness transparently.

### Study selection and evidence-network feasibility

**Figure 1:**
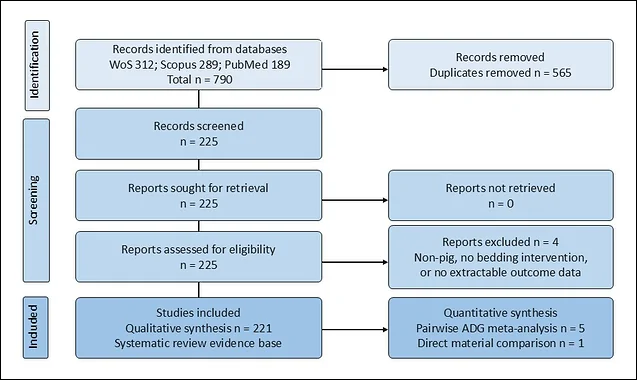
PRISMA 2020 flow diagram of study selection. The diagram summarizes the records identified from Web of Science, Scopus, and PubMed; the deduplicated records screened; the studies included in the qualitative synthesis; and the subsets included in the pairwise meta-analysis and direct material-specific comparison.

The PRISMA flow diagram is shown in Figure 1. The final flow comprised 790 records identified from Web of Science (n = 312), Scopus (n = 289), and PubMed (n = 189); 565 duplicate records removed; 225 reports screened and assessed for eligibility; no reports not retrieved; 4 records removed during final duplicate/eligibility reconciliation; and 221 studies included in qualitative synthesis. Eleven studies contained production-outcome information relevant to performance interpretation, but only five had data adequate for the pooled ADG synthesis [22–25].

**Figure 2:**
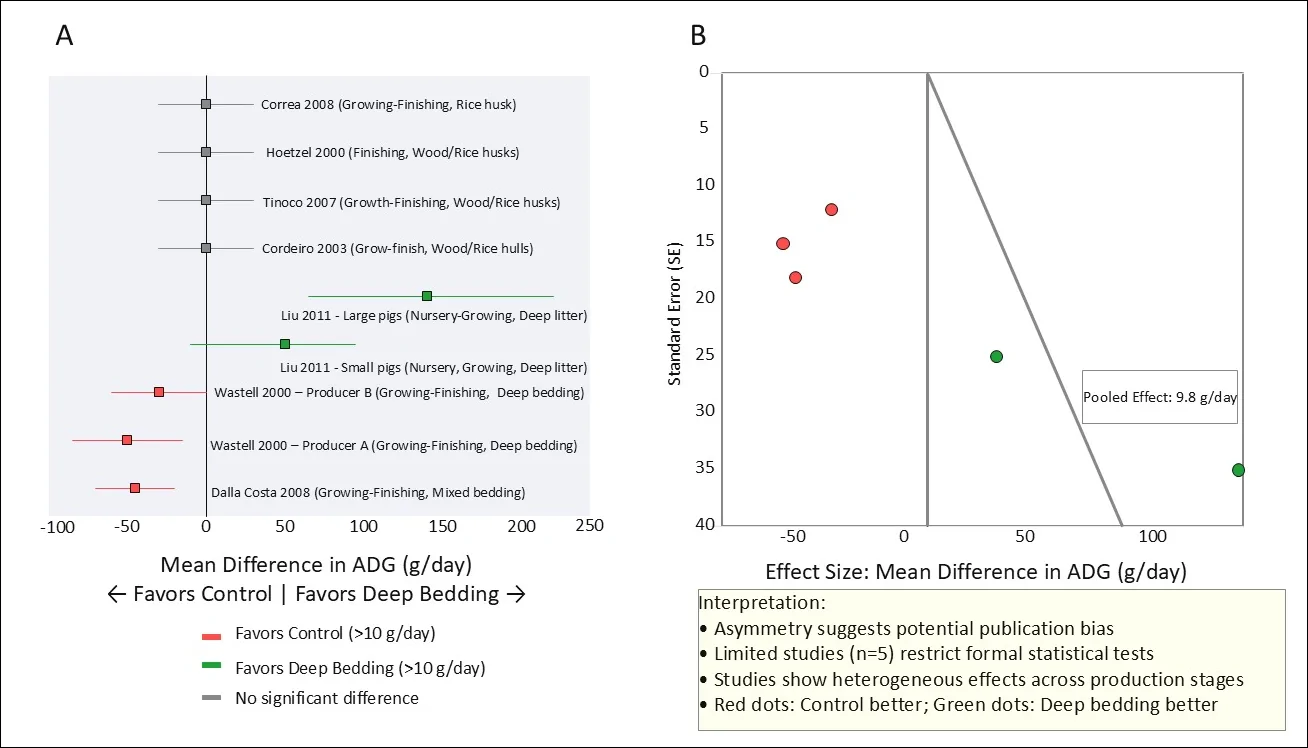
Pairwise meta-analysis of production performance. (A) Forest plot of average daily gain (ADG) comparing deep-bedding with conventional housing. (B) Funnel-plot used to qualitatively assess publication bias. The pooled estimate indicated no significant overall effect despite substantial between-study heterogeneity.

Figure 2 presents the pairwise meta-analysis of production performance. The forest plot comparing average daily gain (ADG) between deep-bedding and conventional housing showed no statistically significant pooled effect, despite substantial between-study heterogeneity (Figure 2A). The funnel plot was visually examined to assess potential publication bias (Figure 2B).

**Figure 3:**
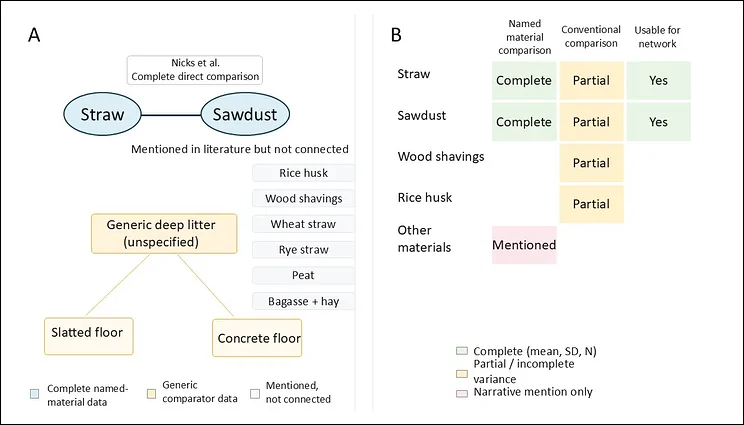
Evidence-network geometry and evidence availability for bedding material comparisons in deep-bedding pig systems. (A) Network geometry showing that only one direct-comparison between named bedding materials (straw vs. sawdust) was available for quantitative analysis. Other materials, including rice husk and wood shavings, were reported but lacked usable head-to-head comparisons. (B) Evidence-availability matrix indicating studies with complete data, partial data, or narrative-only evidence. The disconnected evidence structure prevented network meta-analysis and treatment ranking.

This left the material-specific evidence-network disconnected. Straw and sawdust were linked through a single head-to-head study, but rice husk, wood shavings, mixed bedding, wheat straw, rye straw, peat, bagasse/hay mixtures, concrete floors, slatted floors, and generic deep-litter were either isolated nodes or only partially connected. Figure 3 therefore represents a negative but important finding: the field is not yet structured to support defensible substrate ranking by NMA.

Rice husk illustrates the network limitation and the regional opportunity. It appeared in tropical and subtropical studies as a sole substrate, a depth-manipulated substrate, or a component of mixed bedding, but these studies generally lacked complete head-to-head material comparisons with extractable per-arm means, SD/SE, and sample sizes. Rice husk was therefore synthesized qualitatively in Table 7 rather than ranked quantitatively.

**Table 7:** Focused summary of rice husk evidence in deep-bedding pig systems.

**Refefrence**	**Country**	**Production stage**	**Comparison**	**Main relevance to rice husk interpretation**
[31]	Brazil	Grow-finish	Deep-bedding (wood shavings/rice hulls) vs. concrete	No significant differences in ADG or FCR; higher ammonia emissions in deep-bedding systems.
[32]	Brazil	Finishing (summer)	Rice husks or wood shavings vs. part-slatted concrete	Growth performance and meat quality were broadly similar; bedded pens provided welfare-related behavioral benefits.
[33]	Brazil	Growing-finishing	Deep-bedding (wood shavings/rice husk) vs. concrete	Concrete showed a slight numerical advantage, but overall growth performance was not materially influenced by housing system.
[34]	Brazil	Growing-finishing	Rice husk (0.25 m) vs. rice husk (0.5 m) vs. concrete	Rice husk depth had no adverse effect on growth performance, although litter depth altered the thermal profile.
[29]	Colombia	Finishing	Rice husk deep-bedding vs. concrete	ADG and FCR were broadly similar; mortality was numerically higher in deep-bedding, whereas compost value slightly improved profitability.

ADG = Average daily gain; FCR = Feed conversion ratio.

### Pairwise synthesis of production performance

**Five study/comparison entries contributed to the pooled ADG synthesis:** Dalla Costa 2008, Liu 2011 small pigs, Liu 2011 large pigs, Wastell 2000 Producer A, and Wastell 2000 Producer B. The overall random-effects estimate was MD +9.8 g/day (95% CI –58.3 to +77.9; p = 0.78), with high heterogeneity (I2 = 85.9%; Q = 28.5, p < 0.001; τ² = 3247) and a wide 95% prediction interval (–112.5 to +132.1 g/day). This result indicates no reliable overall production advantage or disadvantage and substantial uncertainty about farm-level effects [26–28].

Subgroup interpretation by production stage helped explain this heterogeneity. Nursery-growing pigs favored deep-bedding (2 study/comparison entries; MD +87.0 g/day, 95% CI +12.0 to +162.0; p < 0.05; I2 = 78%), whereas growing-finishing pigs favored conventional housing (3 study/comparison entries; MD -41.7 g/day, 95% CI –52.4 to –31.0; p < 0.01; I2 = 45%). The between-stage difference was 128.7 g/day (p < 0.05), supporting a stage-specific biological and management interpretation.

Biologically, this stage effect is plausible. Young pigs may benefit from thermal buffering, exploratory occupation, and softer lying surfaces during weaning and adaptation. Heavier growing-finishing pigs, especially in warm or humid climates, may be more vulnerable to heat load, wet bedding, ammonia, and management-related feed or hygiene penalties. The stage-specific trade-off summary is provided in Table 8.

**Table 8:** Stage-specific welfare-production trade-offs and priority evidence gaps identified in the integrated synthesis.

**Stage/context**	**Welfare signal**	**Production signal**	**Main management risk**	**Priority evidence gap**
Nursery- growing	Often favorable: improved exploratory behavior, thermal buffering, and softer lying surfaces.	ADG favored deep-bedding (MD = +87.0 g/day).	Wet bedding, dust, and pathogen load after weaning.	Replicated trials with complete reporting of means, SD/SE, pen-level effects, and standardized welfare outcomes.
Growing-finishing	Behavioral benefits are likely, but comfort depends on heat load and litter management.	ADG favored conventional housing in the pooled subgroup (MD = −41.7 g/day).	Heat load, ammonia accumulation, bedding compaction, and feed wastage.	Climate-stratified trials incorporating production, emissions, and standardized welfare outcomes.
Rice husk/tropical systems	Promising regional substrate for exploratory behavior, moisture management, and circular-resource use.	Evidence was predominantly qualitative and not suitable for NMA.	Litter depth, aeration, dust, compost maturity, and transport cost.	Direct comparative trials linking rice husk, straw, and sawdust under Southeast Asian production conditions.
Breeding/ farrowing	Potential improvements in comfort and nesting behavior, but evidence remains fragmented.	Insufficient extractable production data.	Piglet crushing, hygiene, thermal balance, and labor requirements.	Stage-specific welfare protocols with standardized reporting of sow and piglet outcomes.

ADG = Average daily gain; MD = Mean difference; NMA = Network meta-analysis.

### FCR and mortality

Evidence for FCR was weaker than evidence for ADG. Six studies or comparisons reported FCR-relevant information involving more than 1,000 pigs, but reporting was commonly qualitative, direction-only, or missing complete variance. The GRADE profile therefore treats FCR as a very low certainty qualitative synthesis: results were mixed, with no defensible pooled estimate across the full evidence set [29, 30].

Mortality data were sparse. One extractable comparison (140 pigs) produced an RR of 3.63 for deep-bedding versus conventional housing, but the 95% CI was extremely wide (0.20 to 65.84), so the direction and magnitude of any mortality effect remain very uncertain. Mortality was therefore not interpreted as evidence that deep-bedding is intrinsically safer or riskier; rather, it indicates the need for better event reporting.

### Direct material-specific comparison: straw-versus-sawdust

**Figure 4:**
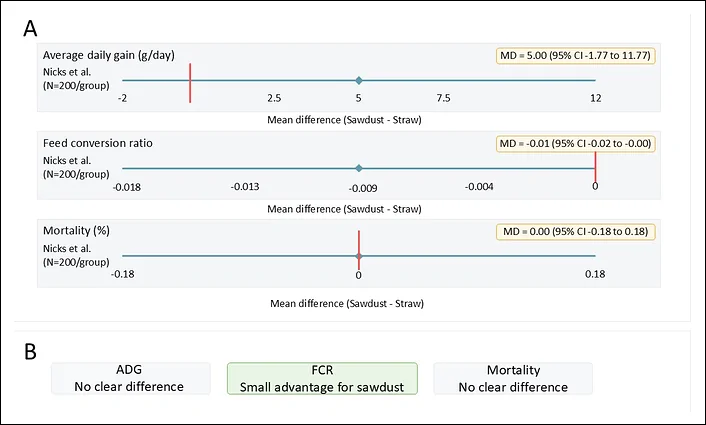
Direct comparison between straw and sawdust in nursery pigs. (A) Effect estimates for average daily gain (ADG), feed conversion ratio, and mortality from the only eligible head-to-head study. (B) Summary of the direct comparison, showing a small advantage of sawdust for feed conversion ratio, with no clear differences in ADG or mortality. As the analysis was based on a single study, the findings should be interpreted with caution and not taken as a treatment ranking.

The only complete direct substrate comparison was the nursery pig study by Nicks *et al.* [30] comparing straw and sawdust. Each group contained 200 pigs, and the duration was approximately 42.6 days. As shown in Figure 4, sawdust had a small, non-significant ADG difference relative to straw (MD +5.0 g/day, 95% CI -1.77 to +11.77; p = 0.148), a statistically small FCR advantage (MD -0.01 kg/kg, 95% CI -0.018 to -0.002; p = 0.0128), and identical mortality (2.5% in both groups; MD 0.0%, 95% CI -0.176 to +0.176). 

Evidence concerning rice husk was broader than the direct-comparison evidence presented in Figure 4, but it was less suitable for quantitative synthesis.Brazilian, Colombian, and tropical/subtropical studies using rice husk alone or in mixtures generally reported performance like concrete or other bedding systems, while emphasizing management-sensitive outcomes such as litter depth, moisture, ammonia, fly burden, compost value, and heat stress. These findings support the use of rice husk as a regionally relevant substrate in rice-producing systems, but not as one that can yet be ranked as superior by formal NMA [31–34].

### Risk-of-bias and certainty of evidence

The ROBINS-I assessment indicated that serious risk of bias was common, particularly due to confounding, selection, missing variance data, and selective outcome reporting. Quantitative studies were often pragmatic farm comparisons rather than randomized pen-level trials, making them relevant to practice but vulnerable to management-bundle confounding.

GRADE certainty reflected these weaknesses. ADG was rated with very low certainty todue of serious risk of bias, very serious inconsistency, imprecision, and suspected selective reporting. FCR was also very low certainty because results were mixed and variance reporting was incomplete. Mortality was very low certainty because the evidence came from a single comparison with an extremely wide CI. The direct sawdust-versus-straw FCR result was more precise but still limited by its reliance on a single study.

### Animal welfare and health synthesis

**Figure 5:**
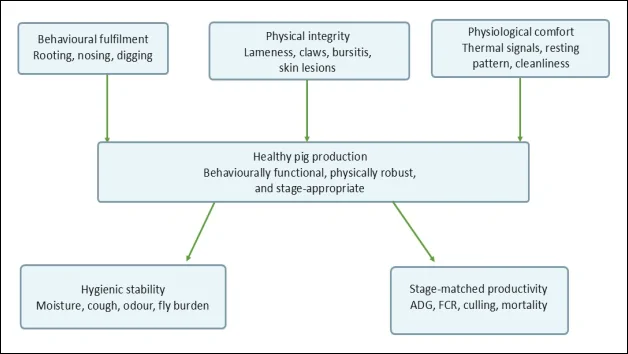
Healthy Pig Production Framework (HPPF) for deep-bedding systems. The framework integrates five interacting domains: behavioral fulfillment, physical integrity, physiological comfort, hygienic stability, and stage-matched productivity. It provides a translational framework for veterinary and farm-level assessment, emphasizing that healthy pig production depends on the interaction among welfare, hygiene, and production performance.

Welfare outcomes were the most consistent findings in the review. Deep-bedding generally increased exploratory behavior, rooting, nosing, substrate manipulation, and occupation with manipulable material compared with conventional non-bedded housing. These outcomes correspond directly to the behavioural and mental-state domains incorporated into the Healthy Pig Production Framework shown in Figure 5 and were more consistent across studies than production-related responses.Physical welfare outcomes were generally favorable when bedding was dry, clean, and sufficiently friable. Several studies reported fewer claw lesions, skin abrasions, bursitis-type injuries, or lameness indicators in bedded systems; however, these benefits declined when bedding became wet, compacted, dusty, or poorly ventilated. This conditional pattern is why the analysis frames deep-bedding as a managed welfare process rather than a static intervention.

Recent Thai evidence is especially relevant to tropical interpretation. Studies from Chiang Mai and related tropical settings suggest that deep bedding can increase exploratory behavior and reduce fly burden under certain management conditions, while concrete systems may offer production advantages when bedding moisture, heat load, or hygiene are not optimally controlled. This supports the stage-, climate-, and management-specific interpretation rather than a universal recommendation.

### Environmental synthesis

**Figure 6:**
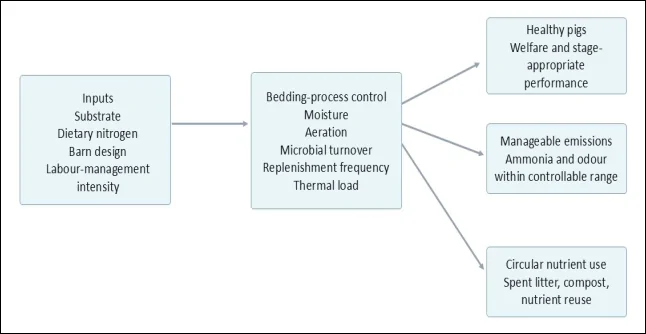
Sustainable Deep-Bedding Model (SDBM) for management-dependent system outcomes. The model links substrate type, dietary nitrogen load, barn design, and management intensity to bedding process control and three principal outcomes: healthy pigs, reduced emissions, and circular nutrient use. Sustainability depends on effective process control rather than on the substrate alone, and on successful litter stabilization, compost recovery, and nutrient recycling.

Environmental outcomes revealed the clearest welfare-sustainability trade-off. Deep-bedding can reduce dependence on liquid wastewater and create a recoverable compost or solid manure stream, which is valuable for mixed crop-livestock systems. At the same time, poorly controlled moisture, nitrogen loading, compaction, and ventilation can increase the risks of ammonia, odor, dust, and nitrous oxide [38–44]. These interacting pathways are summarised in the Sustainable Deep-Bedding Model presented in Figure 6.

Ammonia emissions were often higher in deep-bedding than in conventional housing, especially where dietary nitrogen, bedding moisture, aeration, and replenishment were poorly managed. This does not negate welfare benefits, but it means environmental claims require explicit process-control measures such as dietary protein management, ventilation, dry bedding maintenance, aeration/turning, additives where appropriate, and timely spent-litter removal.

Rice husk carries distinctive environmental implications. Its porosity, silica content, slow decomposition, and regional availability may support moisture control and circular nutrient recovery in rice-producing areas. However, these same properties require attention to litter depth, aeration, dust, compost maturity, and local transport costs.

Greenhouse gas patterns were mixed. Deep-bedding may reduce methane relative to slurry systems by retaining manure in a more aerated matrix, but nitrous oxide can increase when aerobic and anaerobic microsites coexist. Net climate performance therefore depends on bedding moisture, carbon-to-nitrogen balance, stocking density, turning frequency, and manure-use pathway.

### Economic and operational interpretation

**Figure 7:**
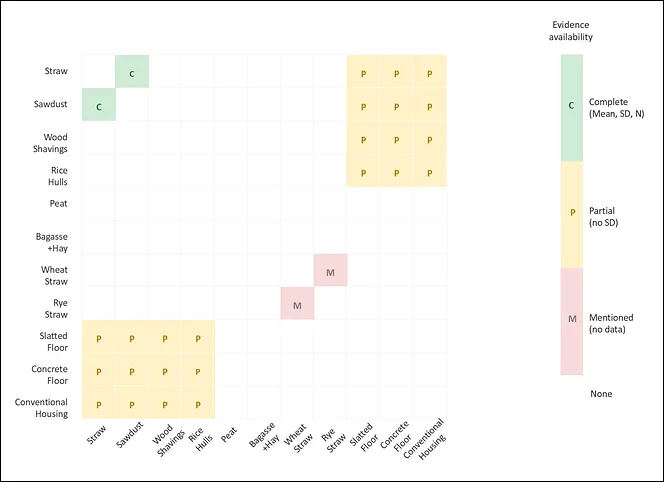
Evidence gap map and future research priorities for deep-bedded pig systems. The map highlights the lack of direct comparisons of bedding materials, limited evidence from tropical production systems and rice husk bedding, incomplete reporting of variance measures, insufficient integration of environmental and welfare outcomes, and the need for validation of the Healthy Pig Production Framework and Sustainable Deep-Bedding Model.

Economic results were mixed and strongly context-dependent. Deep-bedding may reduce liquid-manure infrastructure needs and produce a compostable output, but it also requires bedding purchase or collection, labor, storage space, and management skill. Profitability is most plausible when welfare premiums, local substrate availability, reduced wastewater costs, or crop-nutrient value offset these inputs. Stage-specific welfare-production trade-offs are summarized in Table 8. The main evidence gaps are also visualized in Figure 7, which highlights the need for complete material comparison trials, tropical rice husk experiments, standardized welfare indicators, and complete variance reporting.

## DISCUSSION

### Principal interpretation

The evidence does not support a simple judgment that deep-bedding is uniformly superior or inferior to conventional housing. Instead, the integrated synthesis supports three linked conclusions: welfare responses are more consistently favorable than production responses; production effects differ by production stage and management context; and material-specific substrate ranking is not yet possible because the evidence network is disconnected.

From a production perspective, the pooled ADG result should be interpreted as a context average. The high heterogeneity, wide prediction interval, and divergent stage-specific estimates mean that deep-bedding may be beneficial in nursery-growing settings yet less favorable for heavier pigs under warm, wet, or poorly ventilated conditions. This interpretation is more useful for veterinary advice than alone pooled estimate alone.

### Novelty and originality

This review makes four original contributions. First, it organizes deep-bedding evidence around animal welfare rather than treating welfare as secondary to productivity. Second, it identifies stage-specific heterogeneity, with nursery-growing and growing-finishing pigs showing different ADG signals. Third, it transparently reports the failed NMA as a methodological finding that exposes the absence of connected, material-specific evidence. Fourth, it introduces the HPPF and SDBM as newly developed translational tools for veterinary audit, farm management, and sustainable production planning.

These contributions are especially relevant to tropical and rice-producing regions because rice husk is locally available, potentially circular, and underrepresented in complete comparative trials. To our knowledge, no previous deep-bedding pig review has combined a welfare-centered synthesis, a pairwise production meta-analysis, an explicit material-network feasibility assessment, and two practice-facing frameworks in a single manuscript.

### The HPPF

The HPPF treats welfare-supportive housing as a multidimensional veterinary audit problem rather than a single productivity endpoint. It links behavioral fulfilment, physical integrity, physiological comfort, hygienic stability, and stage-matched productivity to observable indicators such as rooting time, lying behavior, claw lesions, lameness, panting, huddling, body cleanliness, cough, odor, fly density, ADG, FCR, and mortality.

This framework explains why apparently conflicting studies can both be credible. A pen may improve exploratory behavior and resting comfort while simultaneously creating hygienic or environmental risk if moisture and manure load are not controlled. The framework is therefore proposed as a checklist for structured veterinary assessment, not as a claim that any one bedding material automatically improves all domains.

### The SDBM

The SDBM reframes deep-bedding as a managed, biophysical, circular-resource process. Substrate input, dietary nitrogen, barn design, and labor-management intensity influence bedding moisture, aeration, microbial turnover, replenishment frequency, thermal load, emissions, and compost maturity. This framing links animal welfare to manure management and nutrient recovery rather than treating bedding only as enrichment.

This process-based view is central to sustainability claims. Deep-bedding can reduce liquid effluent and support nutrient recovery in crop-livestock systems, but only when spent litter is composted or reused responsibly and when risks of ammonia, odor, dust, and greenhouse gases are actively monitored. The SDBM therefore provides a practical structure for farm audits and for future validation studies.

### Implications for practice, veterinary audit and research design

For producers and veterinarians, deep-bedding is most defensible where welfare improvement, reduced liquid effluent, or manure circularity are explicit priorities, and where daily management can maintain stable bedding conditions. Veterinary audits should assess substrate quality, moisture, compaction, ventilation, nitrogen load, fly density, respiratory signs, lameness, claw lesions, body cleanliness, and stage-specific productivity targets [35–37, 48].

**In terms of research design, the review highlights a persistent weakness:** studies often compare entire housing bundles rather than isolate substrate effects. Future trials should report per-arm means, SD/SE, sample sizes, pen replication, study duration, bedding depth, replenishment frequency, moisture, ventilation, climate, genotype, diet, welfare scores, emissions, and manure-output metrics. Material comparison trials should be designed to connect straw, sawdust, wood shavings, rice husk, mixed bedding, and conventional comparators in a network suitable for NMA.

### Comparison with recent tropical evidence

Recent Thai and broader tropical evidence are especially informative because it tests deep-bedding under warm, humid conditions where wet litter, heat stress, fly pressure, and feed losses may be harder to control. These studies support the idea that welfare benefits and production trade-offs can coexist. Rice husk deserves particular attention in Southeast Asian rice-producing regions because it links local by-product use, bedding availability, and compost return to crop land [49, 53].

This tropical evidence does not invalidate the case for deep-bedding; it defines the management boundary conditions for success. In warm climates, ventilation, stocking density, bedding depth, turning, replenishment, and moisture control are likely to determine whether behavioral and comfort benefits are achieved without unacceptable heat, ammonia, or hygiene penalties.

### Material selection guidance

Material choice is often where farms want a simple ranking, but the present evidence base does not yet support one. The only complete direct comparison suggests a very small FCR advantage for sawdust over straw in nursery pigs, while evidence for rice husk is promising but mostly qualitative. Therefore, a defensible material decision should match substrate properties to farm goals, climate, stage, bedding supply, manure-use plan, and labor capacity.

Recent material-oriented studies reinforce this caution. Bedding materials differ in water-binding capacity, dust, thermal behavior, microbial turnover, pesticide or contaminant risk, pen cleanliness, and fly burden. These differences can affect welfare, product safety, and environmental outcomes, so material selection should be treated as a risk-managed decision rather than a fixed hierarchy [45–47, 50–52].

A practical approach is to use straw where exploratory enrichment and fiber availability are priorities, sawdust or wood shavings where absorbency and feed efficiency are priorities, and rice husk where regional availability, cost, moisture management, and nutrient cycling make it attractive. In all cases, bedding should be managed to remain dry, friable, and compatible with the pigs' production stage and thermal environment.

### Implications for policy and implementation

At the policy level, the findings support welfare-oriented housing reform while cautioning that welfare improvements do not automatically deliver environmental gains. Programs aligned with WOAH guidance and national welfare codes, including updated New Zealand pig welfare discussions on manipulable materials and housing standards [48, 54], should pair substrate provision with monitoring of emissions, hygiene, thermal comfort, and manure management.

The same caution applies to circular-livestock narratives. Deep-bedding can contribute to nutrient reuse and reduced wastewater generation, especially in integrated rice-pig or crop-livestock settings, but these benefits materialize only when spent-litter is composted or applied responsibly and when externalities such as ammonia, odor, and greenhouse gases are controlled.

### Strengths and limitations

This review has several strengths. It uses a welfare-centered structure, integrates production and environmental outcomes, reports quantitative pooling only where data allow, treats failed NMA as informative, identifies rice husk as a regional evidence gap, and develops two practice-facing frameworks that can be tested in future veterinary audit and farm implementation studies.

The limitations are equally important. The quantitative dataset was small; many studies lacked per-arm means, SD/SE, sample size, or duration; risk of bias was often serious; and heterogeneity was high. Formal gray-literature searching, expert contact, meta-regression, and inter-rater reliability statistics could not be fully documented from the available review files. Language and extractability constraints, the March 2026 search cutoff, possible post-search publications, management-bundle confounding, and the unvalidated status of the HPPF and SDBM should be considered when interpreting the findings.

## CONCLUSION

This systematic review demonstrated that deep-bedded pig systems consistently improve behavioral welfare indicators such as rooting, exploratory activity, and resting comfort compared with conventional housing. However, production outcomes were stage-dependent: nursery-growing pigs showed improved ADG with deep-bedding, whereas growing-finishing pigs exhibited reduced performance under certain conditions. Environmental benefits included reduced liquid effluent and potential for nutrient recovery, but risks of increased ammonia and greenhouse gas emissions were evident when bedding moisture, aeration, and management were suboptimal. Material-specific comparisons were limited; rice husk appeared promising for tropical systems but lacked sufficient quantitative data for ranking.

Deep-bedding should be implemented as a managed welfare and sustainability strategy rather than a simple flooring replacement. Veterinary practitioners can use the proposed frameworks to assess suitability for specific farms, balancing behavioral enrichment, thermal comfort, hygiene, stage-specific productivity, and circular manure management. In rice-producing regions, rice husk offers a locally available, potentially sustainable option when litter depth, moisture control, and ventilation are optimized.

Future research should prioritize connected material comparison trials (including rice husk), complete reporting of means, SD/SE, sample sizes, and welfare indicators, climate-stratified studies, longitudinal designs, and validation of the HPPF and SDBM in real-world veterinary audits. Integration of microbiome, emissions, and economic analyses will further strengthen evidence for sustainable pig production.

Deep-bedding offers clear welfare benefits through enhanced behavioral opportunities and comfort, but production and environmental outcomes are highly management-dependent. The HPPF and SDBM provide practical tools for veterinarians and producers to implement and evaluate these systems effectively. By addressing current evidence gaps and adopting context-specific management, deep-bedded systems can contribute meaningfully to sustainable, welfare-oriented pig production worldwide.

## DATA AVAILABILITY

The curated extraction tables, PRISMA flow data, meta-analysis files, GRADE profiles, NMA feasibility files, and screening logs are publicly available through the Open Science Framework repository and the accompanying supplementary materials. The supplementary dataset includes the raw search results, deduplicated records, screening decisions, lists of included and excluded studies with reasons for exclusion, meta-analysis datasets, GRADE assessments, and NMA feasibility files. Because complete raw numerical data were not consistently reported in the original publications, data extraction limitations, missing values, and conversion procedures are fully documented in the supplementary tables for extraction and quality control.

Supplementary Table S1 contains the complete database search strategies. Supplementary Table S2 provides the PRISMA 2020 checklist. Supplementary Table S3 summarizes the characteristics of the included studies. Supplementary Table S4 compares the properties of the evaluated bedding materials. Supplementary Table S5 presents the animal welfare assessment framework. Supplementary Table S6 summarizes the ROBINS-I risk-of-bias assessments. Supplementary Table S7 synthesizes the available evidence for rice husk as a bedding material. Additional supplementary files include the meta-analysis datasets, GRADE evidence profiles, NMA feasibility tables, screening decisions, excluded studies with reasons for exclusion, and detailed framework implementation materials.

## GENERATIVE AI DECLARATION

Artificial intelligence tools were used solely to assist with language editing and manuscript organization. All generated content was carefully reviewed, verified, and revised by the authors, who accept full responsibility for the accuracy, integrity, and originality of the manuscript.

## AUTHORS’ CONTRIBUTIONS

KM and NC: Conceived and designed the systematic review, developed the integrated evidence synthesis framework, interpreted the evidence, drafted the manuscript, and critically revised the manuscript. KM: Supervised data curation. WW, KS, and CA: Interpreted the evidence and critically revised the manuscript. All authors have read and approved the final manuscript.
